# Impact of health insurance on the use of oral health services in the Peruvian population 2015–2019

**DOI:** 10.1186/s12903-024-04441-0

**Published:** 2024-06-12

**Authors:** Jorge Alfredo Herrera Ruiz, Nilthon Pisfil-Benites, Diego Azañedo, Akram Hernández-Vásquez

**Affiliations:** 1 Universidad Científica del Sur, Lima, Peru; 2 Universidad Tecnológica del Perú, Chiclayo, Peru; 3 Centro de Excelencia en Investigaciones Económicas y Sociales en Salud, Vicerrectorado de Investigación, Universidad San Ignacio de Loyola, Lima, Peru

**Keywords:** Oral health, Health insurance, Health services accessibility, Peru

## Abstract

**Background:**

The high treatment cost of oral diseases is a barrier for accessing oral health services (OHS), particularly in low-income countries. Therefore, this study aimed to evaluate the impact of health insurance on the use of OHS in the Peruvian population from 2015 to 2019.

**Methods:**

We conducted a prospective, longitudinal study of secondary data using the National Household Survey (ENAHO) 2015–2019 panel databases, which collected information from the same participants during each of the five years. The dependent variable was the use of OHS in the three months prior to the survey (yes/no). The independent variable was health insurance affiliation (four years or less/all five years). Both were measured by survey questions. Generalized estimating equation (GEE) Poisson regression models with robust standard errors were used to estimate the relative risk (RR) associated with use of OHS.

**Results:**

We included 4064 individuals distributed in 1847 households, who responded to the survey during each of the five years. The adjusted GEE model showed that those who had health insurance during all five years without interruption were more likely to attend OHS than those who had insurance for four years or less (adjusted relative risk [aRR]: 1.30; 95%CI: 1.13–1.50). In addition, we carried out a sensitivity analysis by recategorizing the independent variable into three categories (never/some years/ all five years), which also showed (aRR: 1.45; 95%CI: 1.11–1.89) that participants with health insurance during all five years were more likely to have used OHS than those who never had insurance.

**Conclusion:**

Therefore, in the Peruvian context, health insurance affiliation was associated with greater use of OHS. The panel data used derives from a subsample of consecutive nationally representative samples, which may have led to a loss of representativeness. Furthermore, the data was collected between 2015 and 2019, prior to the onset of the COVID-19 pandemic, and insurance conditions may have changed.

**Supplementary Information:**

The online version contains supplementary material available at 10.1186/s12903-024-04441-0.

## Background

Oral diseases are considered among the most prevalent conditions worldwide, regardless of country income [1]. The Global Burden of Disease Study 2019 (GBD 2019) estimated that this group of diseases affected almost 3500 million people worldwide [2]. In 2019, the number of cases of oral diseases globally exceeded the reported cases of the other 5 most prevalent noncommunicable diseases (mental illness, cardiovascular diseases, diabetes mellitus, chronic respiratory diseases, and cancer) by 1 billion [1]. On the other hand, the estimated number of disability-adjusted life years due to oral diseases was 23 million according to the GBD 2019 [2]. In addition, chronic diseases, such as diabetes or cardiovascular diseases, have risk factors in common with oral diseases [3]. Given their high prevalence and influence on the overall health of individuals, oral diseases represent a major disease and economic burden for health systems and negatively affect the quality of life of individuals.

The management of oral diseases is usually expensive. Even in high-income countries, high cost is a barrier to adequate and timely care for these diseases. A study conducted in Australia reported that although the high cost of dental health is an obstacle to adequate access to health services, health insurance improves access for the population of low socioeconomic level [4]. On the other hand, the treatment and prevention of these conditions in low- and middle-income countries is deficient, particularly in vulnerable populations [5]. In Colombia, in 2013, economic barriers were found to be a major obstacle limiting timely access to oral health services (OHS) [6]. Another Colombian study reported that during the 1998–2005 period, the health inequity index decreased (from 0.295 in 1998 to 0.270 in 2005) as out-of-pocket spending decreased [7].

Health insurance seeks to narrow the gaps in access to health services, mainly benefiting vulnerable populations. According to the World Bank, achieving an economically accessible health service and finding an adequate financing model are priorities for a country’s development [8], since this would eliminate an important barrier of access to health services. In the case of Peru, the strategies applied to improve health insurance began with the Agreement of Political Parties on health in 2005 assisted by USAID [9], which then led to extending the coverage of Comprehensive Health Insurance (SIS) to the entire population as of 2007 [10] and implementing the Framework Law on Universal Health Insurance in 2009 [11], these strategies have, to some extent, improved access to health services for people at the lowest socioeconomic levels. Insurance coverage in the poor Peruvian population rose from 62 to 74% between 2011 and 2015, and also increased from 75 to 81% in the extremely poor population in the same period of time [12]. However, it has been reported that, in some cases, health insurance is not sufficient to guarantee timely access to these services [6]. A prospective cohort study conducted in the United States with data collected from 1997 to 2004 found that in children in need of OHS care, free preventive care was insufficient to remove barriers to OHS use due to the presence of other social factors, such as transport difficulties [13]. However, a prospective study that collected data over 2 years (2005–2007) in Australia reported that health insurance increases OHS visits [14]. Similarly, a cross-sectional study conducted in Colombia in 2014 found that health insurance was a contributing factor to inequity in access to OHS [15].

The 2022 Global oral health status report (GOHSR) stated that the estimated prevalence rates of caries of permanent teeth in people aged 5 years or more, and of severe periodontal disease in people aged 15 years or older in Peru were 38.2% and 19.2%, respectively in 2019 [16]. The Peruvian Ministry of Health reported that caries is the second cause of morbidity in the country in urban and rural areas, with 42.1% and 57.86% respectively [17]. Peru spent between US$ 1 and US$ 10 per person per year on oral health care in 2019, according to the GOHSR [16]. The number of DALYs due to caries in Peru in 2019 for age groups 15 to 44 and 45 to 59 were 6133 and 6511, respectively [18].

Previous studies in Peru that evaluated the association between health insurance and access to OHS at the national level had cross-sectional designs. These studies found that the type of health insurance is one of the factors that determines greater access to oral health in children under 12 years of age [19] and that the level of access to OHS in the population under 12 years of age is low [20]. Nonetheless, the cross-sectional design does not allow an analysis over time and is subject to different types of bias. Besides, both of these studies included only children under 12 years of age which may limit the external validity of their results, on the other hand, our study includes data representative of the adult Peruvian population.

Taking into account that most previous studies that analyzed the relationship between health insurance and OHS had a cross-sectional design and included mostly younger participants, our study aimed to analyze this relationship longitudinally using the panel database of the 2015–2019 National Household Survey [21]. Thus, this study sought to evaluate the impact of health insurance affiliation on the use of OHS in the Peruvian population during the period from 2015 to 2019.

## Methods

### Design and data source

We conducted a prospective longitudinal study of secondary data using information from the National Household Survey (ENAHO) panel 2015–2019. We chose this time period in order to not include the effect that the pandemic may have had on the association between insurance and access to OHS. The databases used were obtained from the National Institute of Statistics and Informatics (INEI) webpage [22] and were the following: SUMARIA-2015-2019-PANEL, Enaho01-2015-2019-100-PANEL, Enaho01A-2015-2019-200-PANEL, Enaho01A-2015-2019-300-PANEL, Enaho01A-2015-2019-400-PANEL, Enaho01A-2015-2019-500-PANEL. The ENAHO panel survey collects information from urban and rural areas in the 24 departments of Peru and the Constitutional Province of Callao. Data was collected by interviews performed by trained field personnel and the same households were visited each year. The sample was selected in a probabilistic, area-based, stratified, multistage and independent manner in each department [21]. The questions are divided into several topics, including household characteristics, characteristics of household members, health, employment and income, ethnicity, among others [23]. The STROBE guide was used to report the results of this study [24].

### Population and sample size

The 2015–2019 ENAHO panel sample included 10,950 households for 2015, the 2016 sample included 12,164, the 2017 sample included 12,038, the 2018 sample included 12,234 and the 2019 sample included 12,637 households. We only included households that responded each year during this 5-year period and who had complete data for all the variables. Therefore, considering the number of households lost in follow-up, the common panel sample in the 2015–2019 period included 1866 comparable households that completed the 5-year follow-up [21].

We calculated the sample size, even though the follow-up sample had losses and may not be highly important. For the present study, the minimum sample size of participants was calculated using the outcome values reported in the study by Teusner et al. in 2012 [14], which explored associations between health insurance affiliation and OHS visits. They found that 71% of the insured participants regularly attended OHS, while 41.5% of the uninsured regularly attended OHS. The sample size calculation was performed in the Online calculator “OpenEpi” for cohort studies using the Fleiss formula with continuity correction and considering a 95% confidence interval (95% CI), a power of 80%, and an exposed/unexposed ratio of 1, which resulted in a minimum sample size of 100 participants (50 exposed and 50 unexposed). In addition, a sensitivity analysis was performed in the statistical package Stata version 17.0, which estimated different sample size scenarios (the ratio in group 1 was 0.415 in all cases and the ratio in group 2 varied between 0.5 and 0.71) taking into account a 1:1 ratio of the outcome in exposed and unexposed. This resulted in sample sizes ranging from 88 to 1078 participants, respectively (Table S1, Supplementary Material). The final sample included in this study exceeded the required estimate.

The household members were considered as the research unit by the ENAHO, as well as household workers living at home, members of a family pension with up to 9 pensioners and persons who were not part of the household but who were in the household during the 30 days prior to the survey. Likewise, the ENAHO excluded members of family pensions with more than 10 pensioners and household workers who resided outside the home [21]. We included 4064 participants, from 1847 households, who responded to the survey during the five years.

For this study we included participants who had complete data for all the variables during all five years.

### Dependent variable

The dependent variable was dental health care in the last three months prior to the survey each year, which was evaluated by means of question 414.6 of the survey: “Did you receive dental and related services in the last three months?”, which had a dichotomous answer (yes/no).

### Independent variable

**Fig. 1:**
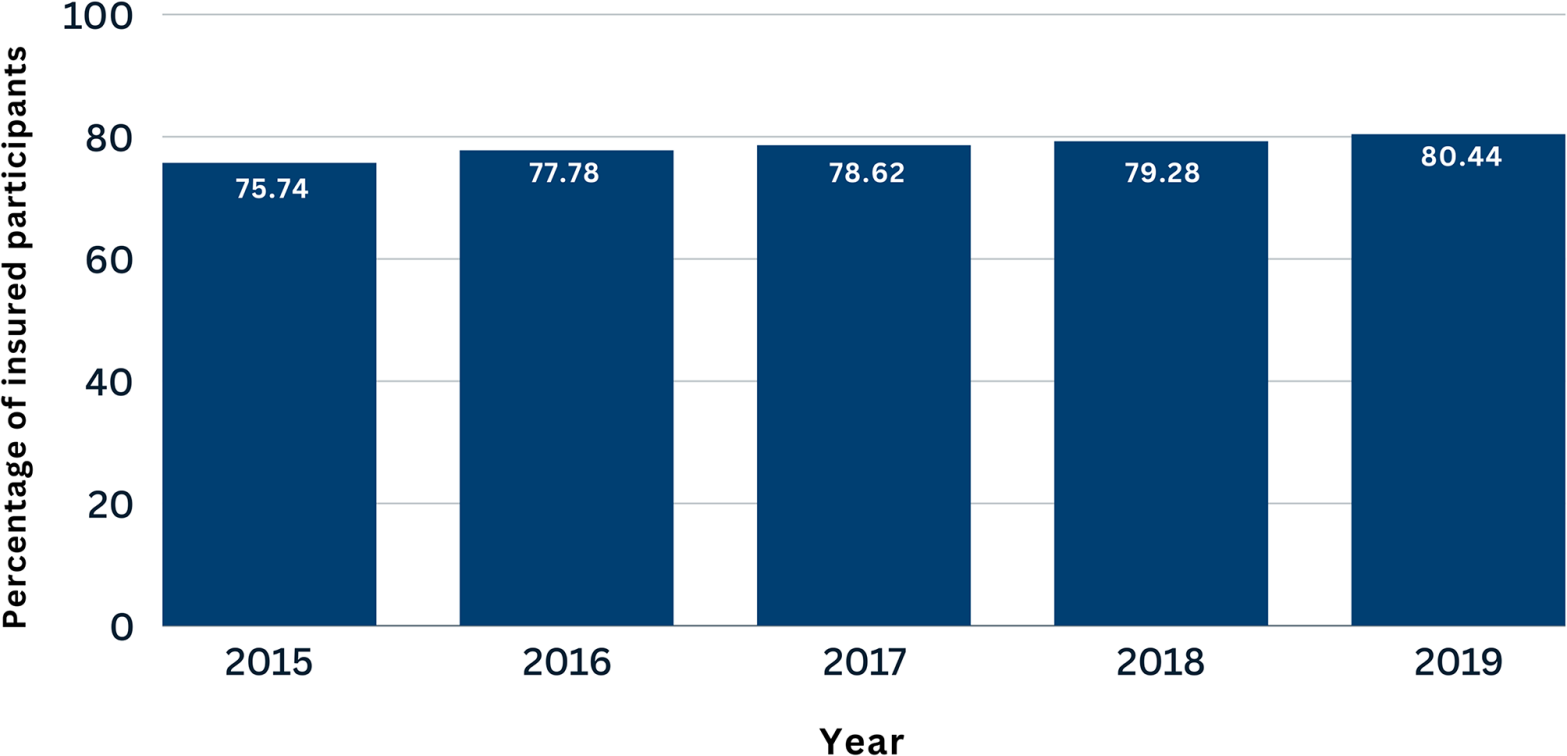
People with health insurance by year according to the 2015–2019 ENAHO panel survey

Insurance status was evaluated by question 419, which states: “The health insurance system to which you are currently enrolled is:”, which had multiple alternatives (types of insurance). A participant was considered as “has health insurance” when they answered “yes” to any of the following 8 options: EsSalud, Private health insurance, Health provider entity, Military/Police insurance, Comprehensive health insurance (SIS), University insurance, Private school insurance, other. Those who answered “no” to all of these 8 options were considered as “does not have health insurance”. Then, the main independent variable was constructed by distributing this information into two categories: “four years or less”, when a participant had health insurance for four or less years, and “all five years”, when a participant had insurance for all five years continuously. Three main entities provide most of the health insurance services in Peru. The Comprehensive Health Insurance (SIS) is mostly aimed at the poor population, is tax-funded and covered 61% of the population as of 2022, EsSalud depends on the Ministry of Labour and provides care through payroll discounts of formal workers, and the Armed and Police Forces insurance depends on the Ministry of Defense and is aimed at military and police personnel, as well as their families; besides, private insurance covers around 10% of the population [26]. We presented the percentage of the population with insurance per year (Fig. 1).

### Covariables

**Fig. 2:**
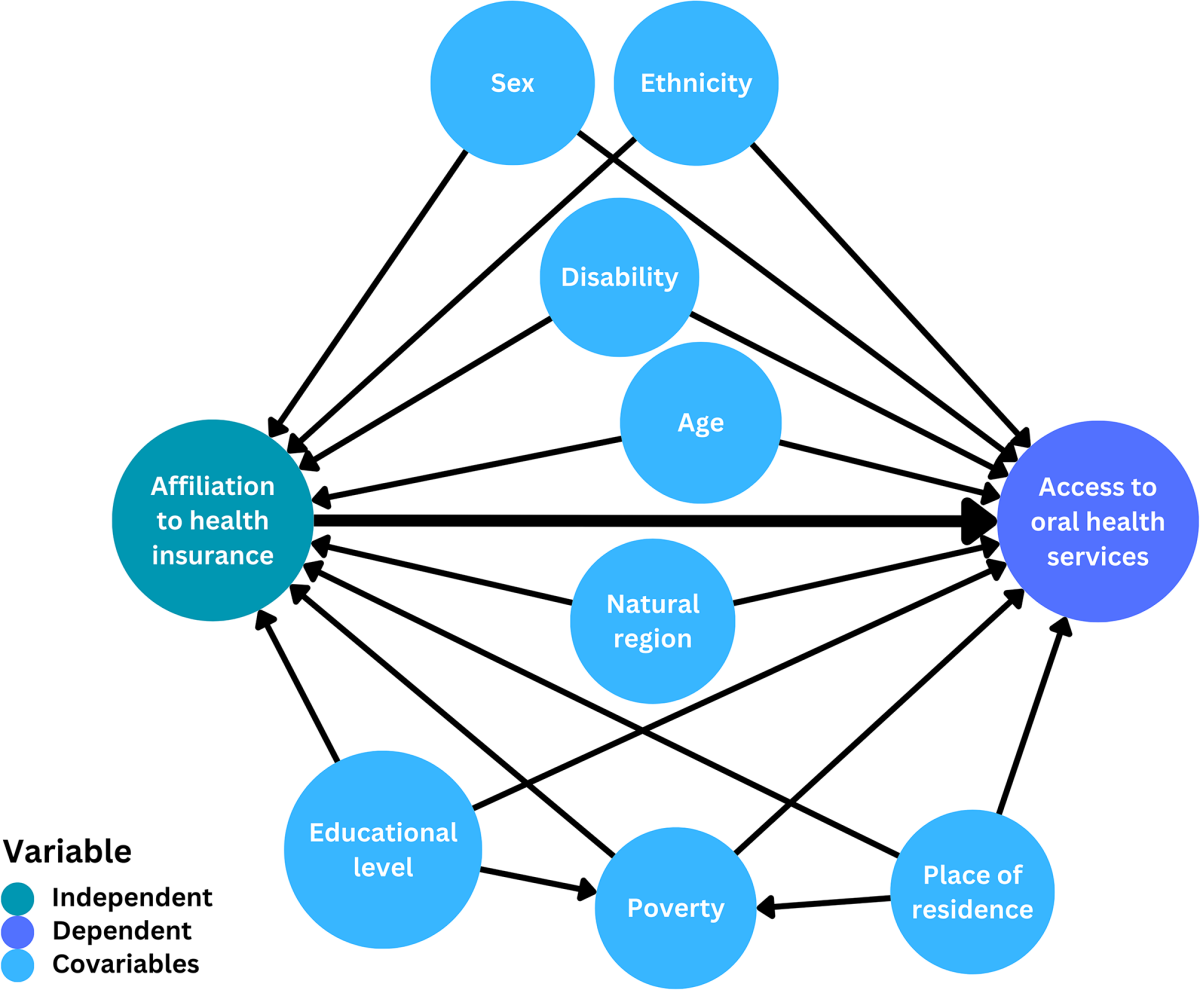
Directed acyclic graph of the relationships between the study variables

Covariables were selected by literature review [25] and by epidemiological criteria. These variables were then used to design a causal diagram to define the relationships between the variables (Fig. 2). The selected covariates were: age group (children: <18 years, young adults: 18–29 years, adults: 30–59 years, older adults > 59 years), ethnicity (native/non-native), household poverty level (poor/non-poor), sex (male/female), educational level (up to primary school/secondary school/higher education), presence of disability (yes/no), area of residence (rural/urban) and natural region where the household is located (coast/highlands/jungle). The level of poverty question in the ENAHO has three options: poor, extremely poor and not poor, this information is calculated from household expenditure and presented as such in the ENAHO database. The data related to this question were dichotomized into poor and non-poor. We considered the poor and extremely poor as poor and the second category was non-poor. The ethnicity variable was categorized as follows: those who identified themselves as Amazonian indigenous, Quechua or Aymara were considered native; and those who considered themselves black/mulatto/zambo/Afro-Peruvian, white, mestizo, other, or doesn’t know were considered non-native.

With the aim of evaluating disability, during the application of the ENAHO, each participant answered a question about whether they had any permanent limitation or difficulty that prevented or hindered them from carrying out their daily activities normally. The response options for this question included: difficulty moving or walking, difficulty using arms or legs, difficulty seeing, even when wearing glasses, difficulty speaking or communicating, even when using sign language or other, difficulty hearing, even when using hearing aids, difficulty understanding or learning (concentrating and remembering) and difficulty relating to others, because of their thoughts, feelings, emotions or behaviors.

Peru has three natural regions, the coast, the highlands and the jungle. The coast is a desert region that extends over a strip of approximately 2250 km in length in which Lima (capital of Peru) and other cities of important economic activity are located. The highlands are a mountainous region located at high altitude (average altitude: 3000 m.a.s.l.) in which the Andes, one of the largest mountain ranges in the world, are located. Agriculture and cattle raising are among the main economic activities in this region. The jungle region is located east of the Andes and contains the Amazon River basin; and is a region with a great variety of flora and fauna whose climate is mostly tropical [26].

### Statistical analysis

The information (database in .dta format) corresponding to the 2015–2019 ENAHO panel survey was downloaded from the microdata repository of the INEI [22].

The databases were then merged and the variables were categorized as previously described. Subsequently, we performed the statistical analysis.

Descriptive analysis was carried out for the first year of the panel survey (2015) (Tables 1 and 2). Qualitative variables were summarized using weighted frequencies including sampling weights, which were added from the ENAHO database. Generalized estimating equation (GEE) Poisson regression models with robust standard errors were used to estimate the relative risk (RR) associated with the use of OHS. RRs and 95% confidence intervals (95% CI) were obtained from the estimated robust standard errors of the model using the *xtgee* command in Stata 17 software (StataCorp LLC, USA). An unstructured correlation structure was used to account for repeated measures at the subject level. The following levels were considered during the analysis: cluster, household, home and individual, as well as the years. A cluster is a grouping of 120 households, which is part of the multistage sampling design of the ENAHO panel survey [23]. First, the crude RR was calculated using the GEE model taking into account the four levels. Then, the adjusted relative risk (aRR) was calculated adjusting for covariates. A *p*-value less than 0.05 was considered statistically significant. The ENAHO sample weights were taken into account during the calculations. Stata SE 17 software was used to perform the analysis.

**Table 1:** Sociodemographic characteristics of the population in 2015 (*n* = 4064)

Characteristics ^a^	*n* (%)
Sex	
Female	2210 (54.38)
Male	1854 (45.62)
Age group	
Children (< 18)	507 (12.48)
Young adults (18 to 29)	687 (16.90)
Adults (30 to 59)	2087 (51.35)
Older adults (> 59)	783 (19.27)
Disability	
Yes	71 (1.75)
No	3993 (98.25)
Place of residence	
Rural	1642 (40.40)
Urban	2422 (59.60)
Household poverty level	
Non-poor	3099 (76.25)
Poor	965 (23.75)
Education level	
Up to primary school	1962 (48.28)
Secondary school	1322 (32.53)
Higher education	780 (19.19)
Ethnicity	
Non-native	3430 (84.40)
Native	634 (15.60)
Natural region	
Coast	1706 (41.98)
Highlands	1542 (37.94)
Jungle	816 (20.08)
Insurance	
Yes	3078 (75.74)
No	986 (24.26)
Use of oral health services	
Yes	409 (10.06)
No	3655 (89.94)

^a^ ENAHO sample weights were included in the descriptive analysis

**Table 2:** Sociodemographic characteristics of the population in 2015 according to the use of oral health services (*n* = 4064)

Characteristics	Use of oral health services	
	Yes *n* (%)	No *n* (%)
Sex		
Female	238 (10.77)	1972 (89.23)
Male	171 (9.22)	1683 (90.78)
Age group		
Children (< 18)	71 (14.00)	436 (86.00)
Young adults (18 to 29)	72 (10.48)	615 (89.52)
Adults (30 to 59)	218 (10.45)	1869 (89.55)
Older adults (> 59)	48 (6.13)	735 (93.87)
Disability		
Yes	6 (8.45)	65 (91.55)
No	403 (10.09)	3590 (89.91)
Place of residence		
Rural	121 (7.37)	1521 (92.63)
Urban	288 (11.89)	2134 (88.11)
Poverty level		
Non-poor	357 (11.52)	2742 (88.48)
Poor	52 (5.39)	913 (94.61)
Education level		
Up to primary school	151 (7.70)	1811 (92.30)
Secondary school	131 (9.91)	1191 (90.09)
Higher education	127 (16.28)	653 (83.72)
Ethnicity		
Non-native	351 (10.23)	3079 (89.77)
Native	58 (9.15)	576 (90.85)
Natural region		
Coast	182 (10.67)	1524 (89.33)
Highlands	135 (8.75)	1407 (91.25)
Jungle	92 (11.27)	724 (88.73)
Insurance		
Yes	335 (10.88)	2743 (89.12)
No	74 (7.51)	912 (92.49)

In addition to the main analysis, we carried out a sensitivity analysis in order to validate our results. For this, we recategorized the independent variable into three categories; “never”, “some year/s”, and “all five years”. Then, similar to the main analysis, we used Poisson GEE regression models with robust standard errors to estimate the RR using the *xtgee* command in Stata 17 software (StataCorp LLC, USA). Similarly, crude and aRR were calculated.

### Ethical aspects

The protocol of this study was approved by the Ethics Committee of the Universidad Científica del Sur with registration code POS-50-2022-00284. The study used a free and open access database from a national anonymous survey conducted by the INEI [21]. The databases we used do not contain personal identifiers. All participants gave consent to participate in the survey.

## Results

We included 4064 people, distributed in 1847 households, who responded to the survey during each of the five years. Table 1 presents the distribution of participants according to the variables evaluated during the first year of the panel sample (2015). Most participants were female (54.38%), lived in urban areas (59.60%), lived in coastal areas (41.98%), considered themselves non-native (84.40%) and had health insurance (75.74%). We found that 2410 (59.30%) participants had health insurance during all five years; 1654 (40.70%) were insured for four or fewer years, and of these, 296 (7.28%) did not have health insurance in any of the five years. Sociodemographic characteristics according to the use of OHS are shown in Table 2.

We found a slight upward trend in health insurance affiliation over the five years. Data shows that the percentage of Peruvians with health insurance has continuously increased by approximately 5% from 2015 to 2019.

On the other hand, the percentage of people that used OHS in Peru was low during all five years. The lowest percentage was reported in 2018 (7.80%) and the highest one was reported in 2015 (10.06%), which shows no apparent increase in this category. But when we analyze only those who used OHS according to whether they had insurance or not we obtained interesting results. In each of the five years, approximately 80% of all people who used OHS had some kind of insurance. The lowest percentage was reported in 2016 (81.38%) and the highest was reported in 2018 (84.23%).

The two types of insurance that were used by the most people during the five years were SIS and EsSalud. The lowest percentage of participants enrolled in EsSalud was reported in 2015 (21.06%) and the highest one was reported in 2019 (23.92%). The lowest percentage of participants with SIS was reported in 2015 (52.41%) and the highest was reported in 2019 (54.50%). None of the other types of insurance reached 2% in any of the five years. In 2015, 30.03% of participants with EsSalud used OHS, and 44.01% of participants with SIS used OHS.

The results of the crude GEE model took into account the four levels, as well as the five years of the panel sample. This analysis showed that individuals who were insured for all five years (RR 1.29; 95% CI: 1.12–1.49; *p* < 0.001) were more likely to attend OHS than those who had health insurance for four or less years (Table 3).

**Table 3:** Generalized estimating equation model to assess the association between having health insurance and having used oral health services

	Crude analysis			Adjusted analysis ^a^		
Variables	RR	95% CI	*p*-value	aRR	95% CI	*p*-value
Insurance						
Four years or less	Reference			Reference		
All five years	1.29	1.12–1.49	< 0.001	1.30	1.13–1.50	< 0.001
Insurance						
Never	Reference			Reference		
Some year/s	1.13	0.85–1.48	0.382	1.14	0.87–1.49	0.336
All five years	1.43	1.01–1.87	0.008	1.45	1.11–1.90	0.006
^a^ Adjusted for age, ethnicity, place of residence, natural region, educational level, disability, poverty level						

CI: confidence interval; RR: relative risk; aRR: adjusted relative risk

The adjusted GEE model analysis is shown in Table 3. This model was adjusted for age, ethnicity, area of residence, natural region, educational level, disability, poverty. After adjusting for covariates, we found that participants who had health insurance during the five years were more likely to visit OHS than those who had health insurance for four years or less (aRR 1.30; 95% CI: 1.13–1.50; *p* < 0.001).

Similar to our main analysis, the adjusted sensitivity analysis showed that individuals with health insurance during the five years were more likely to attend OHS (aRR 1.45; 95% CI: 1.11–1.90; *p* = 0.006) (Table 3).

## Discussion

Economic barriers are an obstacle to have access to OHS. Health insurance emerges as a possible response to narrow the gaps and facilitate the access of the population to these services. Therefore, assessing the relationship between having health insurance and the use of OHS is of great importance for the developers of public health policies. In this context, we conducted a longitudinal analysis taking data from the 2015–2019 ENAHO panel with the aim of evaluating this association in the Peruvian population. Our results show that those participants who had health insurance for all five years attended OHS 1.3 times more than those who were affiliated with health insurance for four years or less.

Defining and comparing the number of individuals who have health insurance among different countries is challenging, due to the great difference among the various insurance regimes that exist in countries of different regions and that may vary over time according to their own conditions. In our study, we found that 59.30% of the population had health insurance during all 5 years; moreover, the number of people with health insurance increased during the period 2015–2019 (Fig. 1). In this sense, the Peruvian health insurance system differs greatly from the system used in Australia, in which health insurance is mainly provided by the private sector, and these services can then be subsidized by the state [25]. A study conducted in Australia reported that in 2008, 50% of the Australian population had health insurance [27]. A study carried out with data from a national survey conducted in children aged 2 to 19 years living in the United States between 2011 and 2014 found that 52.2% of the participants had private health insurance, while 26.6% had public insurance [28]. On the other hand, a study in Colombian adults conducted in 2014 reported that 53.54% of the population had government-subsidized insurance and 35.89% had contributory insurance [15]. Our results show that the number of individuals with health insurance in Peru is similar to other countries in the region, nonetheless, there is room for improvement.

Similarly, the rate of OHS use varies among different countries. In our study, in 2015, 10.06% of the participants attended OHS in the 3 months prior to the survey. However, an Australian study described that 59.2% of the population attended OHS in the 12 months prior to the application of the survey, and the same study reported that the number of insured persons attending OHS increased from 66.3% in 1994 to 69% in 2008 [27]. On the other hand, the study by Azañedo et al. reported that the probability of OHS use in Peruvian children under 12 years of age decreased by 45% between 2017 and 2021 [29]. The use of OHS may depend on several factors that influence the population. In Peru, rural healthcare centers are usually located far from most localities, therefore patients invest more time and money than usual just to access healthcare or even avoid going, due to these barriers. Similarly, transportation systems are inefficient at best and most times they do not exist. Poor and extremely poor population cannot afford the costs related to transportation, particularly when their condition requires multiple visits to healthcare centers. The education level is another important factor since most people do not understand or know the benefits of using OHS, nor do they understand the risks that may incur when not using these services, as suggested by the model proposed by Andersen [30].

Spending on oral health care represents a great economic burden, as shown in the study by Bernabé et al. who, using data from the WHO World Health Survey 2002–2004, reported that households that spent on oral health care were more likely to become poor [31]. In this sense, health insurance is proposed as a solution to narrow the gaps in access to health services. Our results confirm this proposal, showing that health insurance is associated with greater use of health services in the Peruvian population between 2015 and 2019. Similarly, in a study conducted with data from an Australian national survey carried out in 2008, Teusner et al. found that health insurance improved access to OHS in adults of low socioeconomic status, but that the influence had less impact in adults of higher socioeconomic status [4]. A Colombian study conducted in 2014 in adults over 20 years of age concluded that health insurance and educational level are the main factors contributing to reducing inequities in access [15].

However, health insurance alone may not be enough to determine access to health services. Masereijan et al. conducted a study in children aged 6 to 10 years who were followed for 5 years starting in 1997 and reported that free preventive dental health services were insufficient to eliminate disparities in the utilization of such services [13]. Similarly, a study conducted in Canada in 2001 concluded that disparities in oral health status cannot be reduced by the implementation of universal health insurance alone [32]. This may be because health insurance is only one of the factors influencing access to health services.

However, studies conducted in Peru show that health insurance has a positive influence on access to OHS. The study by Hernández et al. reported that the use of OHS increased and inequity decreased from 2004 to 2017, coinciding with the implementation of the SIS in Peru [33]. On the other hand, Azañedo et al. conducted a study in older Peruvian adults in 2018 and found that affiliation to health insurance, among other factors, increased the probability of OHS use [34]. Data from Peruvian studies are encouraging, but more research is needed in order to create enough evidence to help create and implement plans and strategies aimed at the relation between health insurance and OHS.

Previous studies that evaluated the relationship between insurance and the use of OHS carried out in Peru had cross-sectional designs (their results correspond to a single point in time and are subject to more types of bias) and were mainly carried out in children and older adults, not all the Peruvian population. Nonetheless, these types of studies are extremely important as they provide first-hand information on the topic and serve as a basis for future research. On the other hand, our longitudinal study analyzes the relationship between insurance and the use of OHS during a five-year period by interviewing the same persons each year. Longitudinal data, particularly from a nationally representative sample shows a wider picture of how the trend of the relation between insurance and use of OHS has evolved over time. Therefore, our results may be useful when designing public health policies regarding insurance and oral health based on previous trends but aimed at the future.

In 1980, Colombia had a similar insurance model to Peru. It was divided into a private sector, a compulsory social security for workers and employees, and public health services for the poor. Then, in 1993 the General Social Security System for Health was created and included all three modalities; this reduced the state’s role and decreased the competition between different services. Later, in 2003 the WHO, by the world heart report considered that Colombias’ strategy succeeded in providing health insurance to the poor [35]. The insurance system in Peru has improved slowly, despite some deficiencies. Health insurance mostly benefits the poor population, but it doesn’t completely cover all their health needs. Therefore, it may be worth analyzing the overall performance of the system in future studies in order to maybe consider updating the Peruvian insurance model.

Our study has some limitations. Although this was a longitudinal study, we used an existing database, and thus, the information we worked with was not collected specifically for the purpose of this study, which should be taken into account when interpreting our results. Therefore, it is not possible to determine causality due to the nature and design of our study, since some confounding variables may have not been included during the analysis, and it is possible that this may have underestimated or overestimated the effect of health insurance on the use of OHS. It is important to mention that certain variables may have changed over the study period and our analysis did not take into account this variation over time. For example, it is possible that the socioeconomic condition of a household may have changed from one year to the next. Since we used a secondary database, it is possible that errors in data entry may have occurred. Different types of biases may also have occurred during data collection, such as recall bias or politeness bias. Recall and information bias are particularly important, because data was collected from the same people during a five year period, and even the question regarding the recent use of OHS referred to the prior three months, so it is likely that people may have answered differently over the years or even forget important information, which may limit the interpretation of our results In addition, the way in which some variables were measured may present inaccuracies, such as the estimation of poverty or the fact that the use of OHS was assessed only taking into account the three months prior to the survey. Most studies on health insurance and OHS in Peru have been carried out in children or in the older population, therefore the comparison of previous Peruvian studies and our results may incur some bias. Finally, the percentage of data lost to follow-up may have reduced the representativeness of the sample. On the other hand, having used a longitudinal database is a strength of this research, since most studies that address this topic have cross-sectional designs. In this sense, the database we worked with is an official database that is representative of the Peruvian context. However, the data corresponds to the Peruvian population between 2015 and 2019, and thus, our results should be carefully extrapolated to other populations or different years.

## Conclusions

Health insurance is considered to be a way to narrow the gap in access to health services, particularly OHS. In this context, our results show that having health insurance for all five years was associated with greater use of OHS in the Peruvian population evaluated by the ENAHO between 2015 and 2019. In any case, it could be considered that, in order for health insurance to narrow the gaps in access to OHS, it must be accompanied by other measures that seek to solve other problems that may affect the population and generate barriers in access to health services. These other measures could be related to transportation or healthcare infrastructure, among others. Therefore, it would be very useful to carry out studies to evaluate these other factors which, together with health insurance, influence access to health services.

### Electronic supplementary material

Below is the link to the electronic supplementary material.

Supplementary Material 1

## Data Availability

The data used in this study can be accessed freely at https://proyectos.inei.gob.pe/microdatos/Consulta_por_Documentos.asp by selecting “Consulta por encuestas”; then selecting “ENAHO Metodología ACTUALIZADA” as the survey type, “Condiciones de vida y pobreza – ENAHO PANEL” as the survey, “2019” as the year and “2015 Anual – 2019 Anual” as the time period.

## Abbreviations

GBD 2019: Global Burden of Disease Study 2019
OHS: Oral health services
SIS: Comprehensive Health Insurance
ENAHO: National Household Survey
GEE: Generalized estimating equation
RR: Relative risk
INEI: National Institute of Statistics and Informatics
aRR: Adjusted relative risk
95% CI: 95% Confidence interval
