# Supplementary material for: Impact of health insurance on the use of oral health services in the Peruvian population 2015–2019
Source: BMC Oral Health. 2024 Jun 12;24:684. doi: 10.1186/s12903-024-04441-0 (PMC11167752; doi:10.1186/s12903-024-04441-0)
Supplement: Supplementary file 1 — Supplementary Material 1 [file 12903_2024_4441_MOESM1_ESM.docx]

**Impact of health insurance on access to oral health services in the Peruvian population 2015 – 2019**

**Supplementary material**

| **Table S1.** Estimated sample sizes for a two-sample proportions test. | | | | | | | |
| --- | --- | --- | --- | --- | --- | --- | --- |
| **Alpha** | **Power** | **N** | **N1** | **N2** | **Delta** | **p1** | **p2** |
| 0.05 | 0.80 | 1078 | 539 | 539 | 0.005 | 0.415 | 0.5 |
| 0.05 | 0.80 | 228 | 114 | 114 | 0.185 | 0.415 | 0.6 |
| 0.05 | 0.80 | 94 | 47 | 47 | 0.285 | 0.415 | 0.7 |
| 0.05 | 0.80 | 50 | 25 | 25 | 0.385 | 0.415 | 0.8 |
| 0.05 | 0.80 | 28 | 14 | 14 | 0.485 | 0.415 | 0.9 |
| 0.05 | 0.80 | 88 | 44 | 44 | 0.295 | 0.415 | 0.71 |
